# A Treatise on Lesser Surgery, or the Minor Surgical Operations

**Published:** 1835-01-01

**Authors:** 


*A Treatise on Lesser Surgery, or the Minor Surgical Operations.* By BOURGERY, D.M.P. *Translated from the French, with Notes, and an Appendix*, by W. C. ROBERTS and J. B. KISSAM. —New York, 1834. 8vo. pp. 404.

HE is greatly mistaken who supposes that the practice of our profession consists chiefly of interesting or important cases, or who imagines that even the most eminent surgeons are consulted only upon compound fractures and injuries of the head; or that they drive away from performing the operation of lithotomy to a trephining or an amputation: nevertheless, this is a very common error among its junior members. Dazzled by the brilliancy of a capital operation, or seduced by the ingenuity displayed by his teacher in investigating a complicated case, the student of surgery not unfrequently neglects those of daily occurrence, to devote his exclusive attention to what he deems nobler objects of study. Hence the young practitioner is often at a loss upon the most simple points, and his deficiency is apparent when called upon to perform some of those minor operations which are usually entrusted to the general practitioner, or the surgical debutant. Now it must be remembered that life itself may depend upon the dexterity which is shown on these occasions; and, in cases of less importance, a failure in what is thought so simple, makes a most unfavourable impression on the patient's mind; and thus, a man, otherwise of great merit, may be involved in disgrace, lose the opportunity of calling his other and better talents into requisition, and have his best prospects in life clouded, if not altogether destroyed. Or, if he be intended to follow the higher walks of the profession, his attention is no less imperatively required to the minutiæ of his art: for who would trust a surgeon to tie the carotid who was unable to draw blood from the temporal, or to operate for stone when unskilful in the use of the catheter?

Estimating thus highly the importance of minor surgery, it behoves us to inquire how far the present work is calculated to assist the student in this department of his pursuits. We are afraid, but little; not because the author has hurried over his work, nor because he has omitted those minute details which are the cream of his subject, for on these points there remains nothing to be wished for; but because the description of manipulation is extremely difficult to be understood, and, even if perfectly executed, is obviously inferior, as a practical lesson, to witnessing a single performance of the operation in a dexterous manner. For instance, how inefficient would the following description of the mode of bleeding prove to a pupil, though we have no hesitation in saying that, as a description, it is excellent.

" *The Instruments.* The name of the instrument with which bleeding is practised is a lancet, of which three sorts exist, distinguished from each other by the angle made by the point. The obtuse-pointed are called the barleycorn; which form an angle of fifty degrees, and are advisable when a vein is superficial, and we mean to make a large orifice for the issue of the blood. The second kind are called oat-pointed: the angle which the point forms is from thirty-five to forty degrees; they are useful when the vein lies deep; but then, after having made the incision, we must raise the handle of the instrument, to enlarge the wound in the skin, and of the vein itself outwardly. The third kind, the serpent-tongued, are very acute angled, and but little used.

" *The Arrangements.* Bleeding is performed according to general rules. The patient will either sit upon his chair, or will lie upon the edge of his bed. We then proceed to search for the veins in the part wherein we are to bleed, and select always those which are superficial, large, not movable, and not too close to arteries or nerves. Before operating, we are to ascertain the situation of the arteries by the touch, which detects them by their pulsations. In order to distend the veins and make them prominent, the part just above, or below that selected, should be compressed: by the finger, if it is the jugular vein, or if in a limb, by a bandage. We ought not to apply our compression in the latter way so tightly as to impede the circulation through the arteries. We assist in filling the veins by a depending situation of the limb, and by immersing it in tepid water; not, however, to be long continued, lest it might produce redness of the skin, and tumefaction of the cellular tissue, which would render the veins less perceptible. The patient is then directed to cause the muscles below the ligature to contract, and, with the hand applied flatly upon and transversely to the limb, we make graduated pressure, and gently crowd the blood from the branches towards its principal trunk. We generally succeed in a few moments in causing the repletion of the veins; but, if not, we should continue it for half an hour at least before it is abandoned." (P. 158.)

" *Requisite Apparatus.* The things necessary in performing venesection are: first, two good lancets, an obtuse one, and an oat-pointed one, so as to be prepared for any sized vein or situation which we may encounter; secondly, a candle, to afford light, if we are in a dark place; thirdly, a sheet, to spread over the patient, and protect his bed; fourthly, two single-headed roller bandages: one to compress the spot above the vein, before the puncture, and the other to apply over the dressings afterward; fifthly, a compress folded several times, to lay upon the orifice; sixthly, a probe and a pair of dissecting forceps, to remove or put aside globules of fat, should any make their appearance; seventhly, some tepid water, to wash the limb; eighthly, a bottle of eau de Cologne, or of any other volatile stimulating substance, to be used in case of faintness; ninthly, and lastly, two persons as assistants, one of whom will hold the light, and the other the basin.

" *The Operation.* Every thing being now ready, the operator opens the blade of his lancet at an angle of sixty degrees, and places the end of the handle between his teeth, having the shoulder of the instrument turned towards the operating hand; for, as a general rule, we must, except in the case of the arm, bleed from the left side with the right hand, and from the right side with the left hand. He applies the bandage, made of worn linen, by taking two moderately tight circular turns around the limb, at the distance of about two fingers above the place to be opened, and ties it in a bow. One hand is employed in supporting the limb which it grasps, and in stretching the integuments, by means of the fingers on the one side, and of the thenar eminence and thumb on the other, so as to apply the integuments against the corresponding surface of the vein. This tension must be exercised equally on either side, that the same point in the integuments may be over the vein afterward as before they were stretched: if this caution be not attended to, when the skin is let go, after the operation, parallelism is destroyed, and blood is infiltrated into the cellular tissue. Meanwhile, the other hand, by gentle pressure from the branches towards the trunk, drives the blood upwards; the thumb is then lowered to about two inches below the ligature, compresses the vein, now distended with blood, and holds it firm and immovable. The surgeon takes the lancet in his operating hand, holding it betwixt the thumb on one side, and the fore and middle fingers on the other, the ring and little fingers acting as a fulcrum outwardly: by executing a flexory movement, he brings the heel of the instrument into the hollow of his hand, presents the point to the vessel, and then, by a sudden and quick movement of extension, pierces directly to the vein; and, if the lancet be very sharp, or the vessel deep seated, he enlarges the orifice, by elevating its anterior edge and drawing it towards him.

" We very often meet with timid patients, who draw away the limb at the moment when we are prepared to execute the operation. A case like this demands great dexterity on the part of the surgeon, who must endeavour to secure the limb immovably, and, if he cannot succeed in this, must follow the motions of his patient, and avail himself of the first interval of quiet quickly to effect the puncture. The blood spurts out as we withdraw the instrument, and is received in a basin placed for the purpose. It is easy with a little practice to calculate the quantity which issues, which is expressed by the term *palette*, which holds about three ounces. At no bleeding is less than one of these *palettes* detracted, and I have often seen a large bleeding weigh a pound, which is about five *palettes*. When we conjecture that enough blood has been drawn, the thumb of the hand which supports the limb is placed over the orifice, and with the other the ligature is removed; next, we wash the limb with a little tepid water; lay our small square compress, steeped in a solution of salt, over the wound; and secure it by turns of a figure of 8 bandage, with a single-headed roller band." (P. 160.)

Independently of the difficulty of understanding the description of the mode of using the fingers, (though, on reflection, we acknowledge its excellence,) there is one point on which we differ from the author. We object to his method of enlarging the orifice: he employs the lancet as a bistoury, (an error which is very commonly made, but which gives considerable pain,) whereas it is distinctly a puncturing instrument; and, if its point be directed obliquely upwards in the course of the vein, the surgeon may open it as freely as he will, and yet never depart from the proper use of the instrument.

We transcribe also the section upon *Arteriotomy*, as it is short, and gives a good specimen of the manner in which these minor operations are described; and also of the notes affixed to the work by the translators.

"*Arteriotomy*. The necessary conditions for the performance of this operation are, that the artery shall be superficial, of small calibre, and lie over a bone which will allow us to compress it. Several arteries, the temporal, occipital, radial, tibialis anticus where it crosses the instep, &c., answer to these requisites, but, notwithstanding, the operation is performed almost exclusively upon the superficial temporal. It is resorted to in active ophthalmia, but more particularly in apoplexy, phrenitis, and whenever there exists too considerable an afflux of blood towards the brain.

"*Surgical Anatomy*. The superficial temporal artery is the termination of the external carotid. After it leaves the level of the outer angle of the eye, it is situated between the integuments and the temporal muscle. It gives off two branches; the one, posterior, which mounts perpendicularly over the parietal bones, and upon the sinciput anastomoses with that of the opposite side; the other, anterior or frontal, which ascends on the sides of the forehead, and joins its fellow of the other side in an arch. It is this latter anterior branch which is generally opened in arteriotomy.

"*Instruments*. Before we proceed to make a section of the temporal artery, we are to provide ourselves with a bistoury or scalpel, a lancet being likely, from its delicacy of structure, to be broken against the bones of the skull; two graduated compresses, and two bands, one a single-headed roller, the other a double-headed roller, with tails of unequal length, and about six yards long.

"*The Operation*. We first assure ourselves, by the touch, of the position of the artery, which is detected by its pulsations. The thumb and forefinger of the left hand press the vessel against the subjacent bone; in the space between them we introduce the scalpel, the handle of which we hold in our right hand, whilst the forefinger, extended upon the back of the blade, governs and directs its motions. We offer its point to one side of the artery, lower the bistoury, pressing it quite against the bone, and draw it towards ourself, so as to cut the artery crosswise. A red stream, flowing in jerks, which are synchronous with the beatings of the heart, announces the section of the vessel. When it is judged that a sufficient quantity has been detracted, we arrest the flow by making pressure with the finger below the incision, and between it and the heart. We wash the part, substitute one of the graduated compresses for the finger, and lay the other compress over and near to the wound, to guard against recurrent hæmorrhage by anastomosis from the upper end of the vessel. A few tight casts of a bandage generally suffice to control the bleeding. If, however, blood is still discharged, the *packer's knot* bandage must be put on in the following way:

"We apply the flat of the bandage over the graduated compresses; then carry the two heads on the anterior and posterior surfaces of the head; cross them upon the opposite temple, and bring them again over upon the wound. When there, we change hands; the two tails, being one above the other, are strongly drawn downwards, so as to bear upon the compresses, and caused to describe the quarter of a circle by a rapid motion made with both hands in an opposite direction; the one becomes uppermost, and the other the lowest. We then take turns of the bandage vertically around the head, one above the sinciput, the other under the jaw; cross them behind, bring them up again upon the compresses, where, by a fresh knot, their vertical direction is again altered to a horizontal one, and so on until the short head is exhausted; the bandage is then completed by securing the whole by a few circular casts of the remaining globe. In order to guard against a return of the hemorrhage, several days must be allowed to pass over before the dressings are removed.*" (P. 171.)

We must own, if we were to give a description of the method of opening the temporal artery in this country, we should differ considerably from the author. He recommends a transverse section of the artery as a means of bleeding the

* "We supply, by a translation of some parts of the article Arteriotomy, by M. Jules Cloquet, in the new edition of the Dict. de Médecine, Paris, 1833, a few remarks, which will complete the practical usefulness of this section. The posterior auricular artery is also sometimes the seat of operation. The patient either lies upon one side, or, when it is practicable, sits up in a low chair, resting his head, bent to one side, upon the chest of an assistant. The place which had better be punctured is situated at an inch above the zygomatic arch of the temporal and malar bones. An incision three or four lines in length is sufficient for the purpose.

"Small aneurismal tumours sometimes appear afterwards at the seat of injury, but will often spontaneously disperse.—*Trans.*"

patient, but we have long been in the habit of regarding it as a means of arresting the hemorrhage, by permitting the contraction of the circular fibres. The plan which we have been accustomed to adopt, is to make a section of the skin at right angles to the artery, either with a scalpel, or the cutting edge of the lancet, until the vessel is exposed, and then to puncture it longitudinally: by this method the contraction of the circular fibres is rendered useful in separating the edges of the wound. There is another method which we have seen frequently employed, which consists in simply making a very small puncture in the artery, and afterwards applying a cupping-glass. This, though it is perhaps the most painful plan, is attended with one advantage, viz. that the puncture, being exceedingly small, is seldom, if ever, followed by aneurism. Cases will moreover occur where the application of the packer's bandage is productive of pain and inconvenience: in such instances, and in others where it is difficult to stop the bleeding, we should recommend the transverse section of the artery.

In the passages already quoted we have perhaps selected the most objectionable in the book, because they attempt to describe manipulation; but the following observations upon *Leeches*, and their mode of application, are so perfect, that we should find great difficulty in adding anything to them.

"Leeches are kept for use in bottles filled with pure water. The fluid ought frequently to be renewed, twice a week in winter, and in summer every day; neither ought its temperature to be too high or too low; a heat of about fifteen or twenty degrees centigrade best agrees with them. The number of leeches to be put into the bottle must also be proportionate to the quantity of water it will hold: experience has shown that twelve or fifteen of these animals require about a quart of water.

"To succeed in the application of leeches, we must not be indifferent in their selection. Generally those of middle size should be chosen, for it is often difficult to make large ones fix, or they will fall off without having produced any effect: on the other hand, the bite of a very small one is not followed by a sufficient discharge of blood. Another criterion in the choice of a leech is the glossiness of its skin, the strength, swiftness, and flexibility of its movements; and it is seldom that other than a satisfactory issue results from the employment of those thus distinguished.

"Leeches take hold always most quickly of the parts upon which the skin is thin, soft, and lax; and, on the contrary, do not bite at all upon skin which is rough and dry, or covered with a thick and scaly epidermis. For this reason, the skin ought to be placed in a fit condition for their reception, which is done by shaving it, washing it with tepid water, with or without sugar, to moisten and clean it from impurities, and afterwards rubbing it, to produce a determination of blood.*

" *The Application of Leeches.* There are several ways of effecting this object. If one or two only are to be put on, it is sufficient to hold the leech by its hinder end, between the thumb and forefinger, offering its head to the part upon which we wish it to bite. But it is very difficult in this way to make the leech fasten exactly upon the spot we intend: it generally writhes, and essays to get free, or else applies its oval sucker to the fingers which hold it. A long time often elapses before a single leech can thus be made to fasten; and, in consequence of the pressure which it receives, and the fatigue it experiences, its skin dries, it becomes feeble, and rolls itself up into a ball. Several are thus lost unnecessarily, and all the while the affected part is exposed to the cold, which may be attended with serious consequences to the patient. It is therefore, in every case, much better to adopt the following measures, which are particularly suitable when a large number of leeches are to be applied. A number, say six or eight, are collected together in a wineglass, the bottom of which is covered with a piece of paper or a linen rag, to prevent them from sticking; we then turn the glass down upon the skin, on which they soon fasten in a body. The rag should always project beyond the edges of the wineglass, and, when it is reversed, by pulling on the ends of the linen, the leeches are seen lying upon the skin. The number of leeches applied at a single time varies according to the age and strength of the subject, and to the kind, extent, and severity of the inflammation. As many as sixty, and even more, are placed upon the abdomen of an adult, in an acute peritonitis. When, on the contrary, a mere local congestion is to be combated, and particularly in children, one or two leeches may suffice. When put on in such small numbers as this, it is sometimes necessary to make the leech fix upon a part situated in a deep cavity, as the gums, tonsils, lining membrane of the eyelids, neck of the uterus, &c. The necessity for circumscribing the point of suction has given rise to the idea of enclosing the leech, with its mouth placed outwardly, in a hollow tube, open at both ends. A needle-case, or rolled-up card, will answer for this purpose. The cephalic end of the animal is applied upon the spot which is to be relieved; when it has fastened, we set the leech free by pushing on its posterior disk, and drawing off the sheath. Loefler's instrument is constructed upon this principle. Bruninghausen has contrived a similar one, which consists of a glass tube, in which the leech is

* " To the above-mentioned means of making the animal fasten may be added, 1st. Bathing the part with sweetened milk. 2d. Scratching it with a lancet. 3d. Rubbing it with a piece of raw meat, which is sometimes promptly successful. 4th. Allowing the leeches to crawl upon a linen cloth for a few minutes before they are applied. 5th. In winter, letting them swim a little while in warm water beforehand.—*Trans.*"

enclosed, and from which it is expelled by a piston. MM. Brewer and Delaroche caused a hole to be made through the piston, so as to allow of the passage of air. It may be inquired, what advantage is derived from this modification; but to the inventor of the new instrument called the *leech-fixer (pose-sangsues)*, at least, it has not appeared unimportant." (P. 174.)

We here omit the description of the leech-fixer, and the observations of the author on the cause of its success, which he seems disposed to attribute to galvanism. It is so seldom that leeches fail to bite, when proper precautions are taken, that the invention of such an instrument must be looked upon as a work of supererogation. To proceed with their mode of suction.

"The mouth of a leech is of a triangular shape, and has three little jaws or semilunar teeth. These teeth are nothing less than papillæ, armed, according to Dom Allou, with two rows of sixty denticuli each, which, for the three jaws, will make the whole number of little teeth three hundred and sixty. The leech, when it is about to bite, protrudes the semilunar bodies in which the denticuli are, and rounds its head into a disk, which it applies flatly upon the skin, making a vacuum by suction. These motions are produced by the muscles which are attached to the jaws and œsophagus, and by an orbicular one which appertains to the oral sucker. The bite lasts for several minutes, and produces itching, and sometimes very acute pain. A vermicular motion of the leech, by which the rings of its body, from its head to the sucker at its tail, are contracted, announces the commencement of its action. This does not generally go further than the rete mucosum; though it is not uncommon for it to penetrate quite through the cutis. In half or three quarters of an hour the animals have swallowed as much blood as they are capable of containing; they then fall off of themselves, leaving as many triangular orifices, which continue to bleed after their removal. When of a great number of leeches which have been applied a very few only still adhere, it is better to take them off, for they can produce no further effect, and, by remaining suspended by their suckers, they painfully pull upon the seat of injury. This advice will be yet more judicious, when the patient has assumed an inconvenient posture, or there is danger from exposing the part to the cold air. But leeches to be removed must not be torn away, as that might cause the laceration of the small wounds, and subsequently their suppuration; there is a much more simple way, which makes them easily let go; it is to sprinkle upon their heads and backs a little salt, snuff, or lunar caustic, or to pour upon them a little of any irritating solution whatever.

"The quantity detracted by each leech has been estimated at two or three drachms. This approximation is very near the truth, inasmuch as it includes both the blood swallowed by the worm, and that which afterwards escapes from its puncture. It is the opinion of M. Moquin Tandon, that a leech is capable of drawing a quantity of blood which is equal in weight to its own. This assertion has been modified by M. Vernière. According to his experiments, the hirudo officinalis of the smallest size absorbs twice and a half its own weight, or fifty grains; a middle-sized one, twice its weight, or eighty grains: and lastly, the largest sized leech absorbs its own weight, or eighty grains; the medium term of these measurements giving seventy grains, or about a drachm of blood, drawn in by every leech during the period of its suction.

"The quantity of fluid furnished by the bites after the fall of the leeches has also been made the subject of calculation. Some of these wounds bleed for so long a time and in such abundance, that we are obliged to arrest the hæmorrhage; others, again, yield not more than a few drops of blood. In order to estimate the average quantity, the fairest way would be to consider each wound as affording a discharge of blood which is one and a half times the quantity absorbed by the animal itself, or a drachm and a half. Adding this sum to the other, two drachms and a half will represent the weight of blood obtained by every leech, which for every three would be about one ounce. Whence it appears, that to abstract a quantity of blood equal to a venesection amounting to three palettes, we must apply twenty-five or thirty leeches to the patient." (P. 177.)

Then follow some directions as to the method of preventing too great a flow of blood; and the author appears to rely upon compression and styptics. Another excellent means might be added with advantage, viz. the passing a very small thread through the edges of the wound, and thus closing it by a suture. We have employed this plan several times, and have never known it to fail.

From the above quotations, our readers will be able to judge both of the design and execution of the work: it only remains for us to name the various subjects introduced by the author into his book, as, from its elementary nature, it is obviously unfit for any lengthened notice in a Review principally intended for practitioners.

The first section is devoted to the consideration of *Surgical Dressings*, and includes a description of the instruments, compresses, bandages, plasters, splints, &c. used upon such occasions, and a detailed account of the method of employing them.

The second section, on *Topical Medical Applications*, contains chapters on poultices, both emollient and irritating; on baths, general and local, simple and medicated; on fumigations, cerates, and plasters: their composition, mode of employment, and the cases in which they are severally applicable, are stated with perspicuity and brevity.

The third section, which is headed *on Bandages*, but which is rather on *bandaging*, (bandages having been before described,) teaches the various methods of applying them, according to the object in view. It also includes a short account of the various kinds of trusses, which might have been enlarged with advantage.

The next section embraces the *Cutaneous Irritations* and *Artificial Ulcerations*, and the various means of producing them; as caustics, both liquid and solid, setons, the actual cautery, arsenic, blisters, and blistering ointments.

From the fifth section, on the *Simple Operations*, we have already made large quotations. Besides the different methods of drawing blood, it contains rules for making incisions of all kinds, for paracentesis abdominis, for piercing ladies' ears, (an operation in this country performed by the jeweller,) for vaccination, catheterism in the male and female, and the reduction of herniæ. These latter are usually described in elementary works and lectures on surgery, and in many quite as well as by the author.

The book concludes with sections on *Wounds*, *Abscesses*, and *Hemorrhages*, which can scarcely be deemed to belong to minor surgery, but which are nevertheless well treated, as far as the limits and nature of the work permit.

The translators have added an Appendix, which supplies a few omissions of the author, and which does credit to their industry and research. The following passage, which we quote, states a fact that should be known to every practitioner accustomed to perform venesection.

"The blood gushes from a divided artery in a full, rapid, and impetuous stream, which, although alternately elevated and depressed, is nevertheless *continuous*. The blood is of a scarlet hue, unless it issue along with venous blood, which being black, may mask its colour; though, upon close inspection, the separate streams may be detected. If pressure above the wound arrest the flow, the diagnosis is complete. Let it not however be supposed that to these signs any infallibility is to be attached. No jerky stream occurs where the exit of the blood is broken upon the sides of the wound. *A jet of scarlet blood, flowing in jerks, may issue when a vein alone is opened; and uniformly black blood may be poured from an arterial orifice.* In phlebotomy during fever great alarm might very naturally be excited lest the brachial artery had been opened, from the jerky stream and colour of the blood, the result of accelerated circulation, did we not make pressure below the orifice, by which it would, if venous, at once be arrested." (*Appendix*, xxvii.)

We have already expressed our opinion as to the design of the work: in many instances it attempts to teach what can only be taught by actual practice; but, upon other points, it contains much information which cannot be found elsewhere, and which will be useful both to the student, and to such practitioners as have not had the advantage of a very perfect education.

